# The clinical practice of personnel management in performing epidemic containment and treatment tasks for coronavirus disease 2019 (COVID-19) in the county-designated hospital

**DOI:** 10.1097/MD.0000000000044891

**Published:** 2025-09-26

**Authors:** Dongxian Ye, Keqin Ding, Yaner Yu, Chunxia Xu, Yajun Ding, Hongyu Jia, Fang Wang, Libo Zhang, Yingbin Wang, Ludan Xu

**Affiliations:** a Department of the Public Health Care, Beilun Branch of the First Affiliated Hospital, College of Medicine, Zhejiang University, Ningbo, Zhejiang, PR China; b Ningbo Municipal Center for Disease Control and Prevention, Ningbo, Zhejiang, PR China; c Infectious Department, The First Affiliated Hospital, College of Medicine, Zhejiang University, Hangzhou, Zhejiang, PR China.

**Keywords:** code tracing-time division – grading management, COVID-19, designated treatment hospital, personnel control, prevention and control strategy

## Abstract

Sporadic or agglomerated epidemics often occurred in the normalized prevention and control of COVID-19 epidemic in China. This study was the important exploration of personnel management in a county-designated hospital during a regional COVID-19 outbreak. The retrospective research method was used to describe the personnel control measures and infection situation during the treatment of COVID-19 epidemic in stages. Before the policy adjustment, the prevention and control of COVID-19 in China induced the management of class-A infectious diseases. During the regional epidemic outbreak, the regional designated medical institutions of the county-designated hospital implemented the “code chasing time division – grading” procedure of red and yellow code staff, patients, and accompanying nursing personnel and established a personnel management and control system in the hospital. This study analyzed the dynamic data of the personnel control measures and infection situation. This outbreak of COVID-19 started on October 13 and ended on October 26, lasting for 14 days. The personnel with red and yellow codes in the designated hospital underwent a 14 day dynamic-zero process. In a period of rapid growth from October 14 to 20 (days 1–7), there were 173 hospital staffs, 134 patients, and 89 accompanying nursing personnel changed to with the red and yellow codes. From 21 to 27 in October is the period for gradually cleanse for the personnel with red and yellow codes. From 21 to 25, there were 114 hospital staffs, 47 patients, and 20 accompanying nursing personnel with the red and yellow codes changed to the green health codes. Twenty seven patients and 207 accompanying nursing personnel with red and yellow health codes also discharged. On October 27, all groups of the personnel with red and yellow codes were cleared to zero. During the whole outbreak, none of the 3 groups was infected with COVID-19. The “code chasing – time division – grading” procedure implemented in regional designated medical institutions ensured non-battle downsizing of numbers and reduced the unnecessary flow of patients and caregivers in the hospital. This improvement measures for personnel control also provided an experience for preventing and controlling major respiratory infectious diseases in the future.

## 1. Introduction

**Figure 1.**
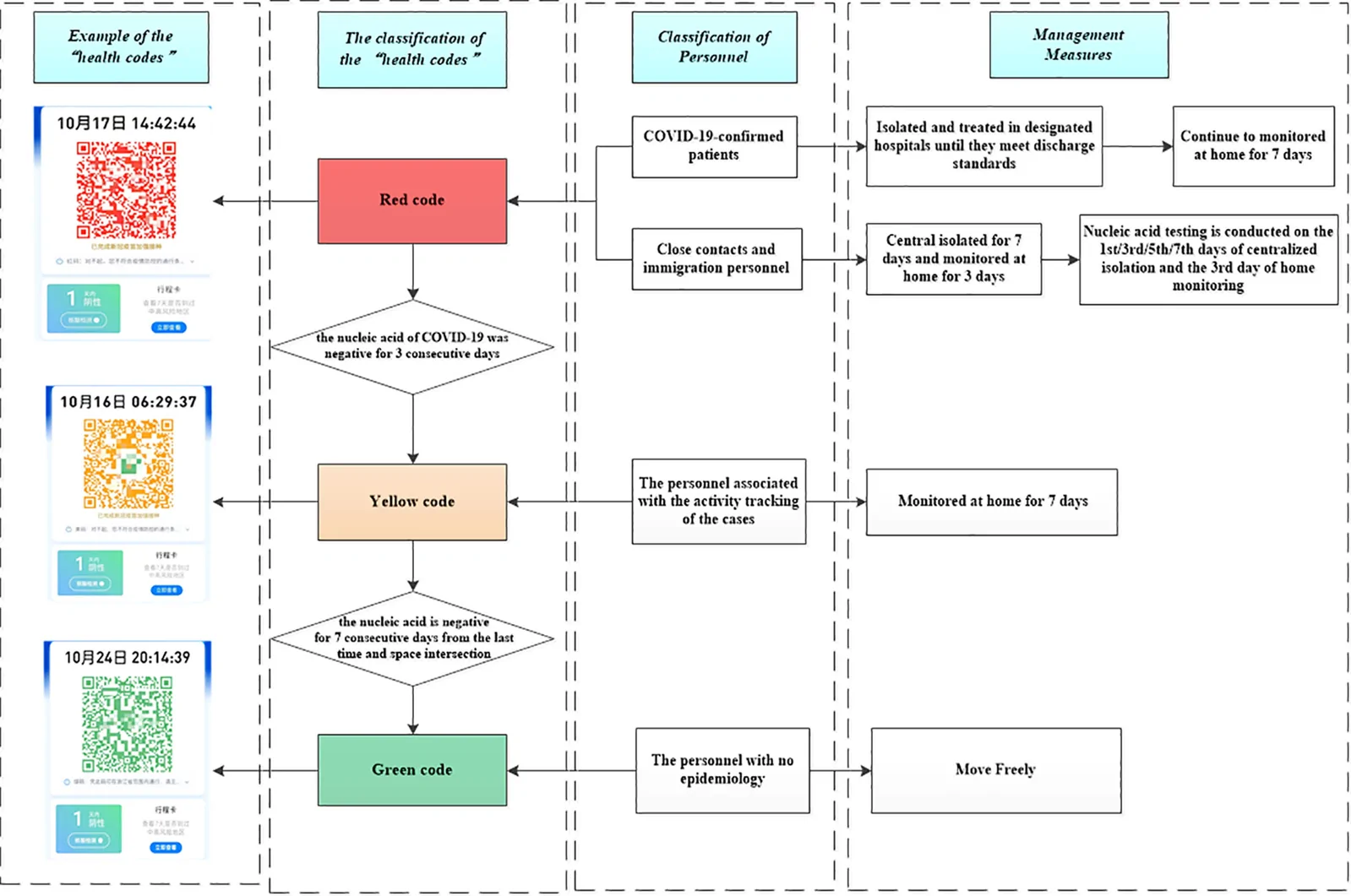
The management of the the personnel with red and yellow codes before the outbreak according to the scheme of Prevention and Control of COVID-19 Infections (Ninth Edition).

At the beginning of the Coronavirus disease-19 (COVID-19) outbreak in February 2020, China, learning from the experience of limiting patients and close contacts mobility confronting the SARS epidemic in 2002 to 2003, launched “mini-apps called “health codes,”^[1]^ which used red, yellow, and green 2-dimensional codes to indicate the corresponding risk level of epidemic transmission and became the basis for judging whether citizens could move freely. The green code is a “basic access facility” in an epidemic society, which is a pass to almost all public places (units, shops, hotels, restaurants, stations, airports, and even parks). According to the scheme of Prevention and Control of COVID-19 Infections (Ninth Edition), COVID-19-confirmed patients, their close contacts, and immigration personnel were assigned red codes. The personnel associated with activity tracking of the cases were given yellow codes, which were changed to yellow codes after the nucleic acid of COVID-19 was negative for 3 consecutive days. The personnel with the yellow codes were changed to the green code after the nucleic acid test was negative for 7 consecutive days from the last contact with the COVID-19 case (see Fig. 1).^[2]^ The wide application of health codes was an innovative measure for managing major public emergencies launched by the government and played a vital role in controlling the spread of the epidemic.^[3]^

In October 2022, an imported outbreak of COVID-19 occurred in 1 district of Zhejiang province. This outbreak was the largest and widest range of COVID-19 cases in the region since the outbreak of COVID-19 in 2020. As a county-designated hospital for COVID-19, the general tertiary hospital was always at the frontline of preventing, controlling, and treating COVID-19. However, as the largest general hospital in the region, critical, emergency, and ordinary patients with other diseases are also treated at this hospital, thus protecting the lives and health of the people.^[4]^

Before the policy adjustment, the prevention and control of COVID-19 in China induced the management of class A infectious diseases. Personnel with red and yellow health codes were isolated at home or centralized. During the disposal process of the local COVID-19 outbreak, hospital staff, patients, and accompanying nursing personnel with red and yellow health codes increased rapidly. Therefore, if all personnel with abnormal health codes are isolated at home or centralized, medical service procedures would be disorderly. To prevent this situation, the command group for the hospital’s epidemic prevention and control took measures according to changes in the pandemic situation. It implemented “code chasing – time division – grading” management, which avoided noncombat downsizing of the hospital staff to ensure medical service procedures. It also reduced the unnecessary turnover of patients and accompanying nursing personnel to ensure medical safety and obtain the overall goal of ending the outbreak within 2 weeks with zero infection in the hospital, achieving the normalization of epidemic prevention and control. We summarized the main practices of prevention and control measures to provide experience for the management of personnel in designated hospitals when dealing with major outbreaks of infectious diseases such as COVID-19.

## 2. Materials and methods

### 2.1. Establishing an information registration platform for sharing information

In the early stages of the imported outbreak of COVID-19, the county-designated hospitals launched a contingency plan. The command group of the hospital’s epidemic prevention and control organized and directed epidemic prevention and control work and made decisions on all staff returning to the hospital on standby. However, with the growth of COVID-19 cases, the number of hospital staff, patients, and accompanying nursing personnel with red and yellow health codes has increased rapidly. The hospital immediately established the information registration system and formulated a unified registration process for personnel with red and yellow health codes; all hospital staff, patients, and accompanying nursing personnel checked the health codes twice daily. If the health codes changed to red or yellow, the appropriate department of the hospital and the community or government control office were contacted immediately to keep up with the superior health department and epidemic prevention department for information sharing.^[5]^ However, after knowing the reasons for the code assignment, the registration of information, including personal information, reasons for the code assignment, assignment time, and brief track since the epidemic outbreak should begin. This information requires accurate, authentic, and real-time update.

### 2.2. Formulating the code-chasing management scheme through accurate research and judgment

**Figure 2.**
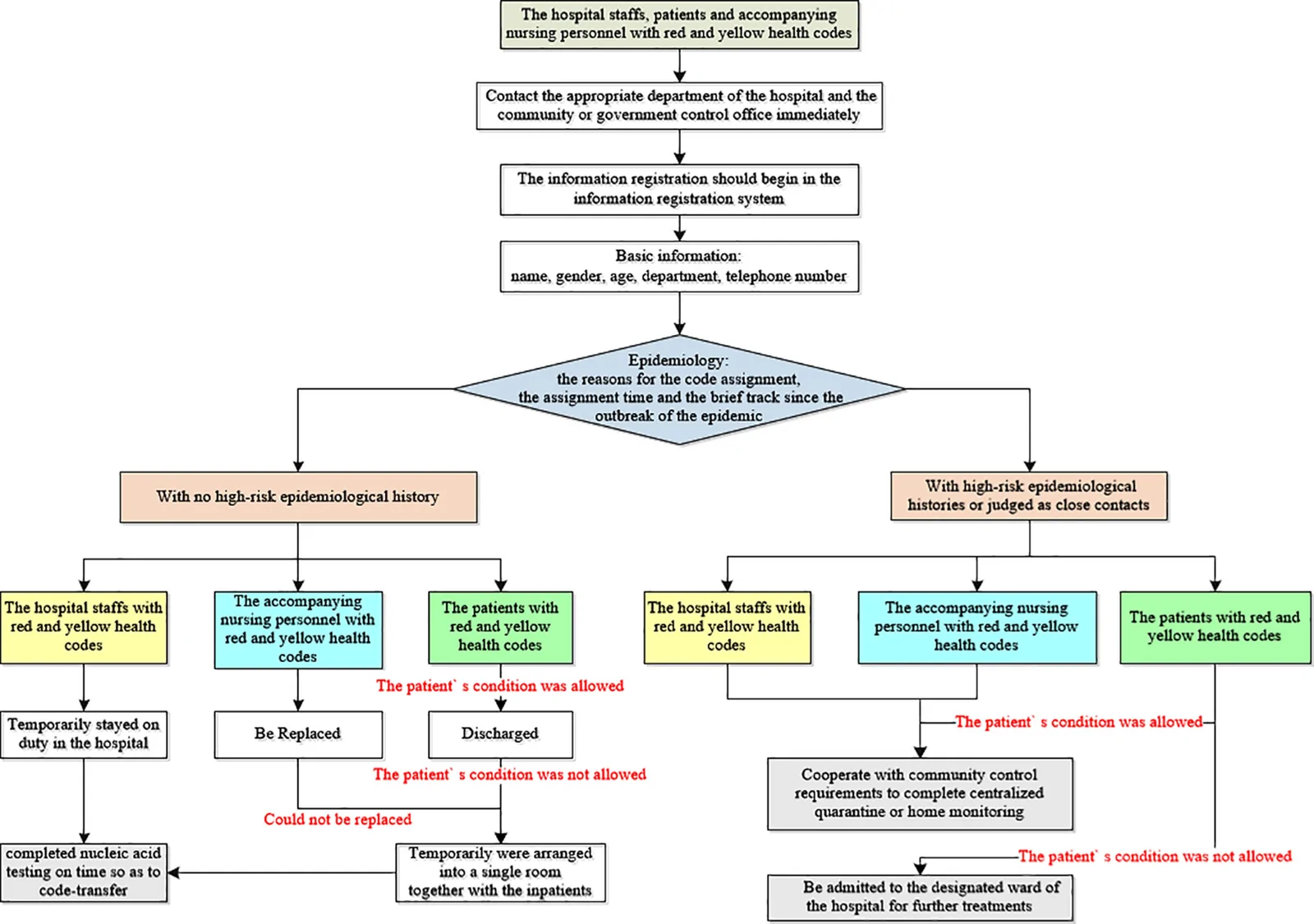
The code-chasing management of the personnel with red and yellow codes during the COVID-19 outbreak.

With the guidance of the epidemic prevention and control command center of the District Health Commission, the command group of the hospital’s epidemic prevention and control formulated the code-tracing management aimed at personnel categories according to accurate research and judgment of the abnormal code. They assigned reasons and brief tracks to personnel with red and yellow codes (see Fig. 2).

#### 2.2.1. The management of the hospital staff

In combination with the reasons and control requirements provided by the community or the government’s epidemic prevention and control system, the hospital staff with red and yellow codes but without high-risk epidemiological histories temporarily stayed on duty at the hospital and completed nucleic acid testing on time to code transfer. Hospital staff with red and yellow codes and high-risk epidemiological histories or judged as close contacts cooperated with community control requirements to complete centralized quarantine or home monitoring.

#### 2.2.2. The management of the patients in the hospital

To reduce the risk of hospital infection, the management of the patients and the accompanying nursing personnel in the hospital were different from that of the hospital staff. In combination with the reasons and control requirements provided by the community or the government’s epidemic prevention and control system, patients with red and yellow codes but without high-risk epidemiological histories were temporarily discharged if their condition allowed or arranged in a single room to complete nucleic acid test on time to code-transfer. If the patient’s condition allows, those with red and yellow codes and high-risk epidemiological histories or those judged as close contacts should be discharged to cooperate with community control requirements to complete centralized quarantine or home monitoring. If the patient’s condition did not allow for discharge, they were admitted to the designated ward of the hospital for further treatment.

#### 2.2.3. The management of the accompanying nursing personnel in the hospital

One inpatient can only be accompanied by 1 nursing personnel in the hospital according to the current epidemic prevention and control requirements. Based on these principles, accompanying nursing personnel with red or yellow codes should be fully replaced. However, if special circumstances exist and the accompanying nursing personnel cannot be replaced, classification management should be performed according to the reasons for the code assignment and control requirements. The accompanying nursing personnel with red and yellow codes but without high-risk epidemiological histories were temporarily arranged into a single room with the inpatients to complete nucleic acid tests on time to code transfer. Accompanying nursing personnel with red and yellow codes and high-risk epidemiological histories or judged as close contacts should leave the hospital to complete centralized quarantine or home monitoring in accordance with the control requirements provided by the community or the government’s epidemic prevention and control system.

### 2.3. Statistical analysis

The regional designated medical institutions provided all clinical data of 198 hospital staff, 174 patients, and 98 accompanying nursing personnel with red and yellow codes during the imported outbreak of COVID-19 in October 2022. Statistical analyses were performed using SPSS version 13.0 (SPSS Inc., Chicago). The paired *T* test was conducted to analyze the time-trend consistency between the number of COVID-19 cases and the the hospital staffs, the patients and the accompanying nursing personnel with red and yellow codes.

## 3. Results

### 3.1. Scientifically implementing and adjusting the control measures based on time division

#### 3.1.1. In the period of the rapid growth of the COVID-19 outbreak (from days 1 to 7)

In the early stages of the COVID-19 outbreak, the number of cases increased rapidly; therefore, there was the possibility of misjudging the red and yellow codes due to the data on the activity trajectory of COVID-19 cases. The command group of the hospital’s epidemic prevention and control tentatively decided that all hospital staff, patients, and accompanying nursing personnel with red and yellow codes in the hospital should temporarily stay in the original ward and perform nucleic acid testing daily after the investigation and judgments. Classification management, according to the code-chasing management of personnel with red and yellow health codes, should be utilized in combination with the reasons for code assignment and control requirements (see Table 1).

**Table 1:** Scientifically implementing and adjusting the control measures based on time division among different people and the periods of the the personnel with red and yellow codes.

The periods of the COVID-19 outbreak	Days interval of the COVID-19 outbreak	Scientifically implementing	The periods of the the personnel with red and yellow codes	Days interval
The period of the rapid growth	Days 1–6	All hospital staff, patients, and accompanying nursing personnel with red and yellow codesshould temporarily stay in the original ward and perform nucleic acid testing daily after the investigation and judgments	The period of the rapid growth	Days 1–7
The period of the stable growth	Days 7–10	The staff with red codes conducted centralized quarantine or home monitoring according to the community control requirements	The period of the rapid clearance	Days 8–12
		The patients with red codes were transferred to a single room in the hospital’s designated ward for further treatment		
		The accompanying nursing personnel with red codes were replaced		
		The hospital staff, patients, and accompanying nursing personnel with yellow codes but without high-risk epidemiological histories temporarily stayed on duty in the hospital and completed nucleic acid testing on time to code transfer		
The period of the gradual clearance	Days 11–14	All hospital staff with red codes cooperated with the community to complete centralized quarantine or home monitoring	The period of the end clearance	Days 13–14
		Patients with red codes are transferred to a single room in the designated hospital ward, whereas those with yellow codes should be admitted to a single room in the general wards		
		The accompanying nursing personnel with red and yellow codes were replaced		

#### 3.1.2. In the period of the stable growth of the COVID-19 outbreak (from days 8 to 11)

After 6 days of extensive data mapping combined with nucleic acid test results, 51.37% of the hospital staff with red and yellow codes and 21.37% of patients and accompanying nursing personnel with red and yellow codes had their codes changed to green (see Table 1, Fig. 2). During this period, personnel with red codes, which did not effectively transform into green, had a high risk of association with the cases. The staff with red codes conducted centralized quarantine or home monitoring according to community control requirements, whereas the accompanying nursing personnel with red codes were replaced. Patients with red codes were transferred to a single room in the hospital’s designated ward for further treatment to reduce the risk of hospital infection. Hospital staff, patients, and accompanying nursing personnel with yellow codes but without high-risk epidemiological histories temporarily stayed on duty in the hospital and completed nucleic acid testing on time to code transfer.

#### 3.1.3. In the period of the gradual clearance of the COVID-19 outbreak (from days 11 to 14)

During the epidemic clearance period, the number of cases decreased, the number of people with red and yellow codes gradually decreased, and the reasons for code assignment were more accurate. All hospital staff with red codes cooperated with the community to complete centralized quarantine or home monitoring according to the reasons for code assignment. Patients with red codes were transferred to a single room in the designated hospital ward, whereas those with yellow codes were admitted to a single room in general wards. Personnel with red and yellow codes cannot be accompanying nursing personnel to further reduce the risk of hospital infection.

### 3.2. The demographic characteristics of the hospital staffs, the patients and the accompanying nursing personnel with red and yellow codes

Among the 198 hospital staffs with red and yellow codes, the age groups were mainly concentrated between 18 and 39 years old and 40 to 59 years old, with females being the majority, accounting for 68.69%. In terms of personnel classification, the number of third-party companies was the most, followed by clinical doctors and nursing staffs, mainly related to personnel flow and total quantity respectively. Among the 174 patients with red and yellow codes, the age groups were mainly concentrated in the age groups of 40 to 59 and ≥60 years old. The majority are males, accounting for 69.54%. From the distribution of departments, the main resources were internal medicine and surgery departments. Among the 98 accompanying nursing personnel, the age group of 40 to 59 years old had the highest proportion, with females accounting for 69.39%. From the distribution of departments, surgery departments was the main resource because surgical patients require accompanying nursing care (see Table 2).

**Table 2:** The demographic characteristics of the the hospital staffs, the patients and the accompanying nursing personnel with red and yellow codes.

The demographic characteristics	Groups	The hospital staffs	Groups	The patients	Groups	The accompanying nursing personnel	*χ* ^2^	*P* value
Age	<18 yr old	0 (0.00)	<18 yr old	10 (5.75)	<18 yr old	0 (0.00)	22.137	.000
	18–39 yr old	104 (52.53)	18–39 yr old	42 (24.14)	18–39 yr old	23 (23.47)		
	40–59 yr old	92 (46.46)	40–59 yr old	54 (31.03)	40–59 yr old	72 (73.47)		
	≥60 yr old	2 (1.01)	≥60 yr old	68 (39.08)	≥60 yr old	3 (3.06)		
Gender	Male	62 (31.31)	Male	121 (69.54)	Male	30 (30.61)	65.42	.000
	Female	136 (68.69)	Female	53 (30.46)	Female	68 (69.39)		
Departments resources	Nursing care	40 (20.20)	Internal medicine	69 (39.66)	Internal medicine	18 (18.37)	1.019	.313*
	Clinical department	56 (28.28)	Surgery	55 (31.61)	Surgery	50 (51.02)		
	Third party company	65 (32.83)	Pediatrics	10 (5.75)	Pediatrics	13 (13.27)		
	Administrative departments	16 (8.08)	Infectious disease department	12 (6.90)	Infectious disease department	1 (1.02)		
	Medical technicians	21 (10.61)	Gynaecology and obstetrics	28 (16.09)	Gynaecology and obstetrics	16 (16.33)		
Total		198 (100.00)		174 (100.00)		98 (100.00)		

* Due to the division of staffs sources by department, patient and accompanying nursing personnel sources are divided by clinical ward. Here we only compared the sources of patients and accompanying nursing personnel.

### 3.3. The changes of the numbers of the hospital staffs, the patients and the accompanying nursing personnel with red and yellow codes

**Figure 3.**
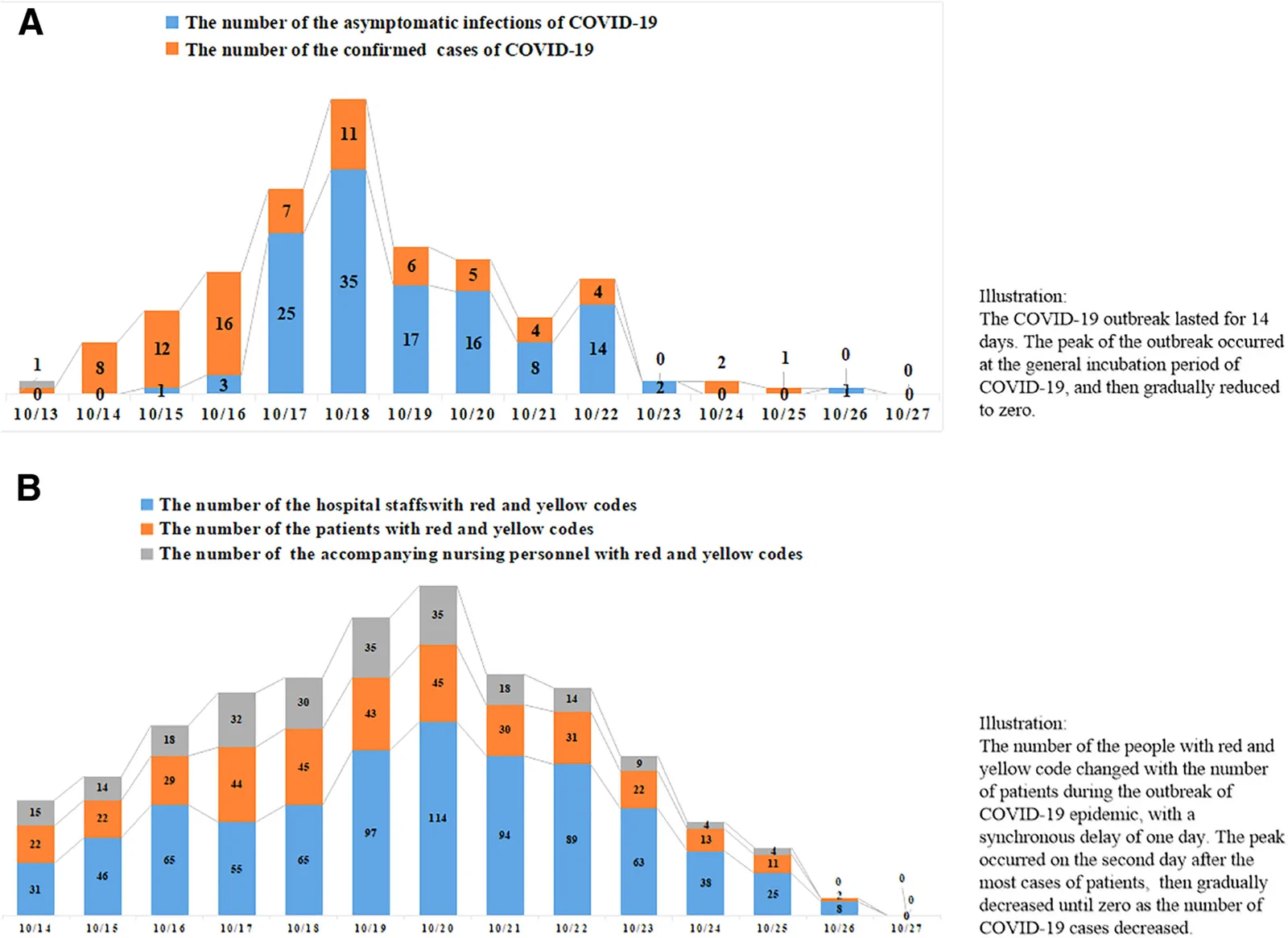
(A) The time distribution of the asymptomatic infections and the confirmed cases during the COVID-19 outbreak. (B) The time distribution of the personnel with red and yellow codes among the hospital staff, patients, and the accompanying nursing personnel during the COVID-19 outbreak.

This outbreak of COVID-19 started on October 13 and ended on October 26, lasting for 14 days. 199 cases of COVID-19 have been reported. From October 15 to 22, the epidemic showed a rapid growth trend, with a total of 184 reported cases (92.46%), followed by a downward trend. The highest number of cases was reported on October 18, with 46 cases (23.11%). The epidemic curve tends to be normally distributed (see Table 3, Fig. 3).

**Table 3:** The changes of the numbers of the the hospital staffs, the patients and the accompanying nursing personnel with red and yellow codes.

Date	The distribute of the cases of COVID-19			The number of the hospital staffs with red and yellow codes				The number of the patients with red and yellow codes					The number of the accompanying nursing personnel with red and yellow codes				
	The asymptomatic infections (1)	The confirmed cases (2)	Total (3) = (1) + (2)	The new number with red and yellow codes (4)	The number changed to green codes from red and yellow codes (5)	The left number with red and yellow codes (6) = (4)–(5)	The secondary infection number	The number with red and yellow codes (7)	The number changed to green codes from red and yellow codes (8)	The discharged number with red and yellow codes (9)	The left number with red and yellow codes (10) = (7)-(8)-(9)	The secondary infection number	The number with red and yellow codes (11)	The number of changed to green codes from red and yellow codes (12)	The discharged number with red and yellow codes (13)	The left number with red and yellow codes (14) = (11)-(12)-(13)	The secondary infection number
10/13	0	1	1 (0.50)	–	–	–	–	–	–	–	–	–	–	–	–	–	–
10/14	0	8	8 (4.02)	31	0	31 (15.66)	0	23	0	1	22 (20.37)	0	19	1	3	15 (22.39)	0
10/15	1	12	13 (6.53)	15	0	46 (23.23)	0	4	0	4	22 (20.37)	0	1	0	2	14 (20.90)	0
10/16	3	16	19 (9.55)	21	2	65 (32.83)	0	13	1	5	29 (26.85)	0	16	3	9	18 (26.87)	0
10/17	25	7	32 (16.08)	30	40	55 (27.78)	0	32	1	16	44 (40.74)	0	25	0	11	32 (47.76)	0
10/18	35	11	46 (23.12)	15	5	65 (32.83)	0	19	2	16	45 (41.67)	0	7	0	9	30 (44.78)	0
10/19	17	6	23 (11.56)	33	1	97 (48.99)	0	14	1	15	43 (39.81)	0	15	2	8	35 (52.24)	0
10/20	16	5	21 (10.55)	28	11	114 (57.58)	0	29	7	20	45 (41.67)	0	6	1	5	35 (52.24)	0
10/21	8	4	12 (6.03)	6	26	94 (47.47)	0	10	15	10	30 (41.67)	0	2	12	7	18 (26.87)	0
10/22	14	4	18 (9.05)	4	9	89 (44.95)	0	26	9	16	31 (28.70)	0	1	1	4	14 (20.90)	0
10/23	2	0	2 (1.01)	1	27	63 (31.82)	0	0	9	0	22 (20.37)	0	2	2	5	9 (13.43)	0
10/24	0	2	2 (1.01)	3	28	38 (19.19)	0	2	11	0	13 (12.04)	0	2	4	3	4 (5.97)	0
10/25	0	1	1 (0.50)	11	24	25 (12.63)	0	2	3	1	11 (10.19)	0	2	1	1	4 (5.97)	0
10/26	1	0	1 (0.50)	0	17	8 (4.04)	0	0	6	3	2 (1.85)	0	0	4	0	0 (0.00)	0
10/27	0	0	0 (0.00)	0	8	0 (0.00)	0	0	1	1	0 (0.00)	0	0	0	0	0 (0.00)	0
10/28	0	0	0 (0.00)	0	0	0 (0.00)	0	0	0	0	0 (0.00)	0	0	0	0	0 (0.00)	0
Total	122	77	199 (100.00)	198	198	0 (0.00)*^,^†	0	174	66	108	0 (0.00)*,†	0	98	31	67	0 (0.00)†	0

* Compared to the distribute of the cases of COVID-19, the distribution of the persons with red and yellow codes was significant.

† Compared with each other group, the distribution of the peasons with red and yellow codes was significant.

The personnel with red and yellow codes in the designated hospital underwent a 14 day dynamic-zero process (October 14–27), with a 1 to 2 day delay in the outbreak of COVID-19. During a period of rapid growth from October 14 to 20 (days 1–7), 173 hospital staff, 134 patients, and 89 accompanying nursing personnel changed to red and yellow codes, with component ratios of 87.37% (173/198), 77.01% (134/174), and 90.82% (89/98), respectively. During this period, by performing nucleic acid testing daily after the investigation and judgments, 59 hospital staff, 12 patients, and 7 accompanying nursing personnel with red and yellow codes were changed to the green code. Seventy seven patients and 47 accompanying nursing personnel with red and yellow health codes, respectively, were discharged according to the personnel management scheme. The number of personnel in the 3 categories with red and yellow codes peaked on October 20. From 21 to 27 in October is the period for gradual cleansing of personnel with red and yellow codes. The number of new personnel with red and yellow codes gradually decreased. From the 21st to the 25th, there were 114 hospital staff, 47 patients, and 20 accompanying nursing personnel with red and yellow codes changed to green health codes. 27 patients and 207 accompanying nursing personnel with red and yellow health codes were discharged. On October 27, all groups of personnel with red and yellow codes cleared to zero. During the outbreak, none of the 3 groups of people was infected with COVID-19.

## 4. Discussion

Coronavirus disease-19 (COVID-19) is a new type of coronavirus that has caused a global pandemic. The disease is highly contagious, and all people are susceptible to it. Therefore, extensive measures have been taken to prevent the spread of the disease in communities and hospitals.^[6]^ Personnel with red and yellow health codes were managed according to the scheme of Prevention and Control of COVID-19 Infections (Ninth Edition) to reduce the risk of COVID-19 infection. During this local outbreak of COVID-19, the regional designated medical institutions implemented the “code chasing – time division – grading” management of red and yellow code staff, patients, and accompanying nursing personnel.

First, the command group of the hospital’s epidemic prevention and control established and operated the information registration platform. WeChat and the DingTalk platform were fully used for database input information. Information registration, analysis, and judgment of personnel with red and yellow codes require professional knowledge support, keen insight, and high ideological standing.^[7]^ In the process of personnel control in this round of prevention and control, control information was obtained through the community, government prevention and control office, epidemic prevention and control information system, and other platforms, providing effective help in formulating personnel control measures.

Second, the hospital carried out scientific implementation of “Precise Control.” According to the activity tracking of personnel with red and yellow codes and big data information on control requirements, implementing code chasing and hierarchical management in different periods of the outbreak can maximize human resources to prevent and control COVID-19, reduce unnecessary floats of patients in the hospital, and identify and isolate personnel with high-risk histories at an early stage^[8]^ to prevent nosocomial cross-infections. All activities of the personnel with red and yellow codes were limited to their original departments, and their diet distribution was centralized to eliminate the float of personnel and reduce the risk of transmission.^[9,10]^ The ward should be strictly disinfected daily with regular ventilation (at least twice daily for >30 minutes each) and disinfection of public goods, living places, household garbage, medical waste, and the excrement of isolated patients according to requirements^[11]^ simultaneously.

Third, all hospital staff, patients, and accompanying nursing personnel with red and yellow codes should check the health code daily, undergo nucleic acid testing daily, and fill in the information in the registration system, such as the time of change to the green code and departure from the hospital. The personnel for centralized isolation or home monitoring, with reference to the isolation point and community management, used the information registered through the WeChat platform to record the detailed health monitoring, nucleic acid detection results, and the end time for control to complete the closed-loop management and grasp the real-time information of the personnel with red and yellow codes to facilitate the next control measures.

The personnel with red and yellow codes in the designated hospital underwent a 14 day dynamic-zero process (October 14–27), synchronized with the dynamic-zero strategy of the outbreak of COVID-19 (October 13–26) to decrease the number of cases and deaths of COVID-19.^[12]^ During a period of rapid growth of personnel with red and yellow codes (days 1–7), 87.37% of hospital staff, 77.01% of patients, and 90.82% of accompanying nursing personnel changed to red and yellow codes, respectively. During this period, personnel with red and yellow codes remained in their original wards. Through the analysis and judgment from the community, the government prevention and control office, or the epidemic prevention and control information system, 59 hospital staff, 12 patients, and 7 accompanying nursing personnel with red and yellow codes were changed to green codes, respectively. 77 patients with an allowed state of illness and 47 accompanying nursing personnel with red and yellow health codes were discharged successively according to the personnel management scheme. From 21 to 27 in October is the period for personnel cleansing with red and yellow codes. The number of new personnel with red and yellow codes gradually decreased. During this rapid cleanse for personnel with red and yellow codes (from 21st to 25th), the personnel with red codes had a higher risk of association with cases. Therefore, the staff with red codes conducted centralized quarantine or home monitoring, nursing personnel with red codes were replaced, and patients with red codes were transferred to a single room of the original ward. The hospital staff, patients, and accompanying nursing personnel with yellow codes, but without high-risk epidemiological histories, temporarily stayed on duty. There were 114 hospital staff members, 47 patients, and 20 accompanying nursing personnel with red and yellow codes changed to green health codes. During the period of end clearance for personnel with red and yellow codes, accompanied by a decrease in the number of cases, the reasons for code assignment were more accurate. All personnel with red and yellow codes were strictly managed according to the scheme of Prevention and Control of COVID-19 Infections (Ninth Edition). Different risky people implemented different measures to reduce the risk of COVID infections and achieve zero infection for the personnel in the hospital.

Through this practical approach, we found that information construction and data collection must be improved. The main limitations were: the database project was not perfect in the early stage; therefore, the later stage needed to fill in the relevant information to obtain related epidemic information; the data collection was not timely because the data collection was manual through departmental levels, and some departments did not execute timely; the objects of this study were 3 categories of people with red and yellow codes according to the epidemiological history with the COVID-19 cases. It is difficult to calculate the sample size, nor to conduct random sampling. In addition, if the epidemiological history is provided inaccurately, there may be classification errors. Therefore, a modern information approach is required to establish a data information registration system that can be sent to each employee’s mobile phone to ensure accurate and timely input.

## 5. Conclusion

The control measures for personnel with red codes requiring centralized isolation and those with yellow codes requiring home isolation were formulated according to China’s epidemic prevention and control requirements at that particular time, which may not necessarily apply to other prevention and control systems.

However, during this outbreak of COVID-19, there was no similar practice for reference in controlling personnel with red and yellow codes in other regions. The command group of the hospital’s epidemic prevention and control performed comprehensive research and implemented the “code chasing – time division – grading” procedure innovatively to prevent the phenomenon of “one-size-fits-all” control of the personnel and to achieve the purpose of “zero input and infection.” Taking the prevention and control of this outbreak as a guide, the hospital summarized concrete measures to further optimize the personnel control system to avoid non-combat downsizing and reduce the risk of nosocomial infection, which is an important exploration for the precise prevention and control of COVID-19. It also provides information for the prevention and control of major respiratory infectious diseases in the future.

## Acknowledgments

All authors acknowledge that there was no financial interest in or benefit from the direct application of this research.

## Author contributions

**Conceptualization:** Chunxia Xu, Hongyu Jia, Yingbin Wang.

**Data curation:** Keqin Ding, Hongyu Jia, Fang Wang, Libo Zhang.

**Formal analysis:** Ludan Xu.

**Investigation:** Fang Wang.

**Resources:** Libo Zhang.

**Software:** Libo Zhang, Yingbin Wang.

**Supervision:** Yaner Yu, Chunxia Xu.

**Validation:** Ludan Xu.

**Visualization:** Yajun Ding.

** Writing – original draft:** Dongxian Ye, Yaner Yu.

**Writing – review & editing:** Hongyu Jia, Yingbin Wang.
